# Photoplethysmography Signal Analysis for Optimal Region-of-Interest Determination in Video Imaging on a Built-In Smartphone under Different Conditions

**DOI:** 10.3390/s17102385

**Published:** 2017-10-19

**Authors:** Yunyoung Nam, Yun-Cheol Nam

**Affiliations:** 1 Department of Computer Science and Engineering, Soonchunhyang University, Asan 31538, Korea; ynam@sch.ac.kr; 2 Department of Architecture, Joongbu University, Goyang 10279, Korea

**Keywords:** healthcare, smartphone, photoplethysmography (PPG), motion artifact, ROI

## Abstract

Smartphones and tablets are widely used in medical fields, which can improve healthcare and reduce healthcare costs. Many medical applications for smartphones and tablets have already been developed and widely used by both health professionals and patients. Specifically, video recordings of fingertips made using a smartphone camera contain a pulsatile component caused by the cardiac pulse equivalent to that present in a photoplethysmographic signal. By performing peak detection on the pulsatile signal, it is possible to estimate a continuous heart rate and a respiratory rate. To estimate the heart rate and respiratory rate accurately, which pixel regions of the color bands give the most optimal signal quality should be investigated. In this paper, we investigate signal quality to determine the best signal quality by the largest amplitude values for three different smartphones under different conditions. We conducted several experiments to obtain reliable PPG signals and compared the PPG signal strength in the three color bands when the flashlight was both on and off. We also evaluated the intensity changes of PPG signals obtained from the smartphones with motion artifacts and fingertip pressure force. Furthermore, we have compared the PSNR of PPG signals of the full-size images with that of the region of interests (ROIs).

## 1. Introduction

Mobile healthcare applications using smartphones have been developed exponentially in recent years and are now an essential part of the daily lives of people in various environments. Mobile healthcare applications can collect health-related information using a smartphone via body-worn or implanted sensors, and can deliver health-promoting messages in situations and throughout people’s everyday life [1]. The use of smartphones is attracting more attention in healthcare. Mobile healthcare applications make smartphones useful tools in the practice of evidence-based medicine at the point of care, in addition to their use in mobile clinical communication [2]. In addition, smartphones can play a very important role in patient education, disease self-management, and remote monitoring of patients.

Smartphones have fast microprocessors, multiple sensors, large data storage, software flexibility, and media capabilities. In other words, they are suitable for developing monitoring systems that can potentially be used by the general public [3]. They have accordingly been found to be accurate in a variety of vital sign monitoring applications [4,5,6,7]. Recently, our research group proposed an approach using dual cameras consisting of contact and noncontact video monitoring directly implemented on a smartphone for estimation of heart rate (HR) and the mean respiratory rate (RR), respectively [8].

Smartphone devices can not only record PPG signals with a camera and a flashlight, but also serve as an accurate monitor for several physiological variables, based on their ability to record and analyze the varying color signals of a fingertip placed in contact with its optical sensor. However, there are some issues in acquiring PPG signals from a camera on a smartphone [9]. First, different smartphone models lead to different color saturation in the captured frames; second, shifting the finger with respect to the camera lens creates motion artifacts and, as a result, causes wrong segmentation; third, different fingertip pressure force on the camera lens as well as different features of the tissue change the level of light absorption when it passes through the finger, thereby causing different color ratios.

Selection of the optimal region of interest (ROI) is one of important issues in the collection of clean PPG signals. The quality of the PPG signals depends on the properties of the skin area, camera characteristics, light emitting diode (LED) usage, and light conditions [9]. Of particular importance is the area of a person’s skin in one or more image frames that should be analyzed, and an ROI should be defined to obtain strong PPG signals. To achieve this goal, Optimal ROI Selector (OptROIS) not only searches for the most stable skin region, since stable regions generally provide the strongest PPG signals, but also selects the optimal ROIs to record clean PPG signals. Thus, OptROIS should analyze spatial pixel uniformity inside the ROI and the strength of a PPG signal. In order to select the location of optimal ROIs that contain strong and clean signals. The obtained images are converted from YUV420sp to RBGA. Then, these sampled signals are filtered with a band-pass filter. After selecting a color channel [6], the signal is interpolated at 30 Hz via a cubic spline algorithm to obtain a fixed sampling rate. Finally, peak-to-peak intervals (PPI) are derived from interpolated signals.

In our previous work [4,5,6], for the video recordings of Samsung Galaxy S3, iPhone, iPad, and iPod, four different pixel region sizes were examined for determining the optimal signal quality. In these works, a larger pixel resolution did not necessarily result in a better signal quality. In fact, in most scenarios, a 50 × 50-pixel resolution was just as good as or better than an HVGA resolution. In addition, the closest region to the flash was in most cases associated with higher signal quality, which is both logical and expected. Finally, using the optimum pixel size, location, and color band of a pulsatile signal (PS), accurate heart rate and respiratory rate can be estimated.

Recently, two other methods were developed to improve the reliability. In [10], after binarizing each frame, the radius of the fitting circle was calculated. In [11], a frame adaptive ROI was proposed to handle the color saturation and cut-off distortion caused by the limited dynamic range of smartphone cameras. However, it focused on improvement of the signal quality rather than reducing the extraction time. Both of these methods had a time-varying ROI, which was confusing due to the time-varying intensity of pixels. In this paper, we show experimental results using three different smartphones to obtain reliable PPG signals, and compare the PPG signal strength in three color bands when the flashlight was both on and off. We also evaluated the intensity changes of PPG signals obtained from smartphones with motion artifacts and fingertip pressure force.

The rest of this paper is organized as follows. The proposed method is explained in Section 2. In Section 3, the implementation of the proposed method and its performance are presented. Finally, conclusions are given with discussions in Section 4.

## 2. Method

### 2.1. Data Collection

Data were collected on 30 healthy subjects (18 males, 12 females) with yellow skin color using three different devices: a Samsung Galaxy S6, a HTC One M8, and a Samsung Galaxy Note 3. The room temperature was kept stable at approximately 22 °C, so that the subjects were comfortable. Soonchunhyang Hospital’s Institutional Review Board approved the data collection technique. For the pulsatile signal (PS) acquisition, Java was used to develop the Android smartphone on the mobile platform Android 4.1 (Jelly Bean). Specifically, we used Eclipse Integrated Development Environment (IDE) Indigo R2 as an integrated development environment for development and debugging purposes. For the video recordings, we examined 8 × 8 ROIs for determining the optimal signal quality. The PS was obtained by averaging the entire pixel size for each of the three color bands (red, green, and blue) for every frame. All three devices provided a sampling rate close to 30 frames per second. However, when the video sampling rate was lower than 30 Hz, a cubic spline algorithm was used to interpolate the signal to 30 Hz.

**Figure 1:**
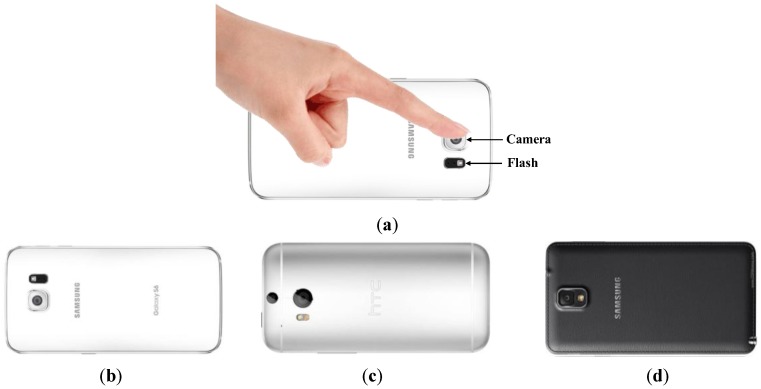
Smartphones used for evaluating performance: (**a**) General scheme to acquire videos from three devices; (**b**) Samsung Galaxy S6; (**c**) HTC One M8; (**d**) Samsung Galaxy Note 3.

No subject had cardiorespiratory pathologies. All three devices were tested using the same subjects, at the same location, and under the same test conditions. Data were collected in the sitting upright position, and the sensor was placed in proximity to the subject’s left index or middle finger. Figure 1 shows data collection on the three devices by placing a fingertip on the video camera.

Having established that the green color signal provides the best signal amplitude values for smartphones [5], we systematically investigated which pixel regions of the color bands gave the optimal signal quality, as determined by the largest amplitude values for all three devices. As shown in Table 1, 15 different conditions were investigated for comparing PPG signal quality.

Thirty seconds of data were collected for each subject. A set of these 15 conditions was repeated five times in the same order. Each 30-s condition was separated by 10-s rest periods. When the LED flash is turned off, we observed that signals are weak and contain more white noise under weak (60 watts, fluorescent lamp, 120–210 lux) light.

### 2.2. Data Analysis

Most smartphone image sensors capture at 30 Hz or 60 Hz. More recently, a new image sensor is capable of 240 fps video capture at full 1080 p resolution. Owing to the frame rate variability, we interpolated the PS to 30 Hz using a cubic spline algorithm followed by peak detection. The peak detection algorithm incorporated a filter bank with variable cut-off frequencies. To extract the experimental data, we set the window size to 60 samples with 50% overlapping segments between consecutive windows at a 30 Hz sampling frequency.

To measure the intensity change of signals obtained from a smartphone camera over time, the change of intensity $i^{*}$ is calculated using the following equation:

(1) $$i^{*}=\frac{\sum_{j=1}^{n}arg\;\underset{k}{\max}\left[x_{ppg}\left(k_{j}\right)\right]-\underset{k}{\min}\left[x_{ppg}\left(k_{j}\right)\right]}{n},$$

where $x_{ppg}\left(k_{j}\right)$ is signal in window *j*.

## 3. Experimental Results

### 3.1. PPG Signal Quality Analysis

**Figure 2:**
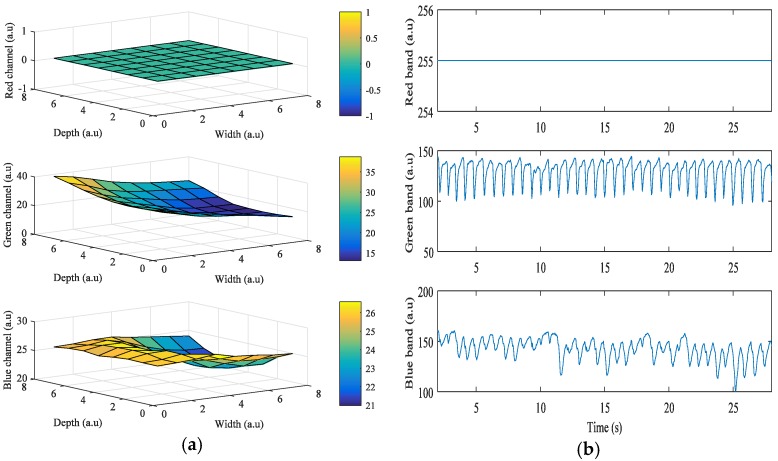
Example of quality analysis of PPG signals obtained from a subject using the Galaxy S6: (**a**) intensity change distribution; (**b**) intensity of PPG signal.

We investigated the signal quality among the red, green, and blue color bands for each pixel region for all three devices. Figure 2a shows the results obtained from the Galaxy S6 using a grid size of 8 × 8. In the figure, the x-axis and y-axis are depicted in grid scale to improve visualization of the intensity (z-axis) of the images when the flashlight was on. Even though the LED flash is placed on the right of the camera, Android device’s orientation is set to landscape mode while acquiring signals. In this case, the right side of the portrait mode ROI is turned to be on top, and the left side is on the bottom. As the ROIs get closer to the flashlight, $i^{*}$ of the green and blue color bands gets higher. However, in Figure 2a, $i^{*}$ of the red color band in the ROIs is 0 when close to the flashlight because the intensity values are always 255, which is the maximum value of intensity. Figure 2b shows the intensity of the three color bands over time when the flashlight was on. In the figure, the average values of $i^{*}$ of the red, green, and blue color bands are 0, 38.7, and 26.59, respectively. We found that the green color consistently provided significantly higher mean amplitude values than either the blue or the red color. We also found that the region closest to the flash in most cases resulted in a higher signal quality, which is logical and expected.

**Figure 3:**
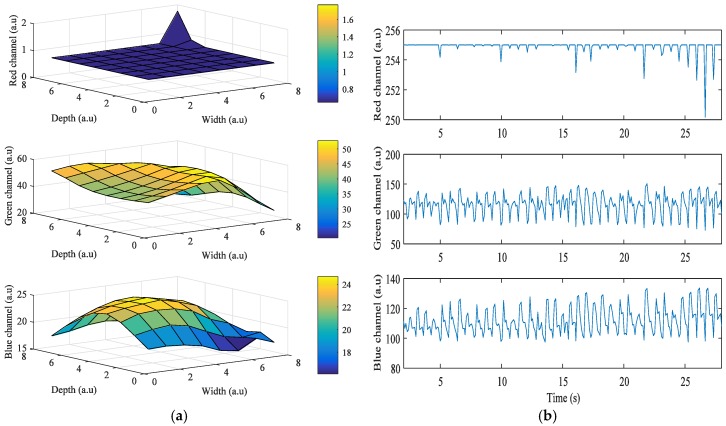
Example of quality analysis of PPG signals obtained from a subject using the HTC One M8: (**a**) intensity change distribution; (**b**) intensity of PPG signal.

Figure 3 shows the results obtained from the HTC One M8, when the grid size was 8 × 8. In this case, the LED flash is placed on the left of the camera, and Android device’s orientation is set to landscape mode while acquiring the signals. The right side of the portrait mode ROI is turned to be on top, and the left side is on the bottom. As the ROIs also got closer to the flashlight, $i^{*}$ of the green and blue color bands also got higher. However, in Figure 3a, $i^{*}$ of the red color band in ROIs when close to the flashlight is 0 because most intensity values are 255, which is the maximum value of intensity. Figure 3b shows the intensity of the three color bands when the flashlight was on. As shown in the figure, average values of i^*^ of the red, green, and blue color bands are 1.77, 52.75, and 24.69, respectively.

**Figure 4:**
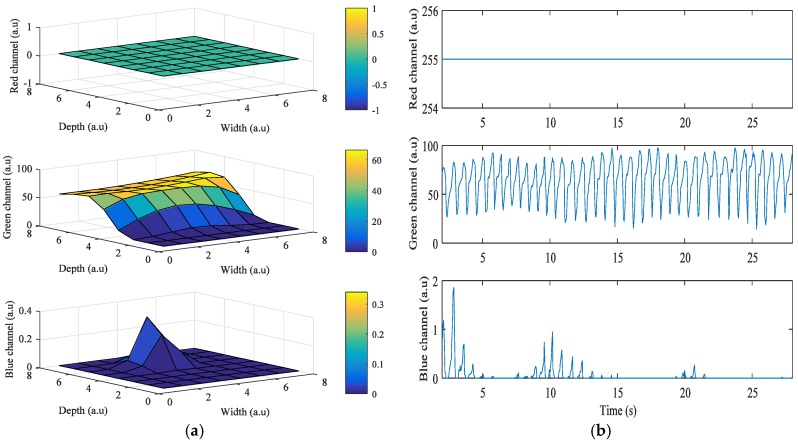
Example of quality analysis of PPG signals obtained from a subject using the Galaxy Note 3: (**a**) intensity change distribution; (**b**) intensity of PPG signal.

Figure 4 shows the results obtained from the Galaxy Note 3 when the grid size is 8 × 8. In this case, the LED flash is placed on the bottom of the camera, and Android device’s orientation is set to landscape mode while acquiring the signals. The right side of the portrait mode ROI is turned to be on top, and the left side is on the bottom. As ROIs got closer to the flashlight, i^*^ of green and blue color bands also got higher. However, in Figure 4a, $i^{*}$ of the red color band in ROIs when close to the flashlight is 0 because the intensity values are always 255, which is the maximum value of intensity. Figure 4b shows the intensity of the three color bands when the flashlight was on. In the figure, average values of $i^{*}$ of the red, green, and blue color bands are 0, 66.38, and 0.34, respectively.

**Figure 5:**
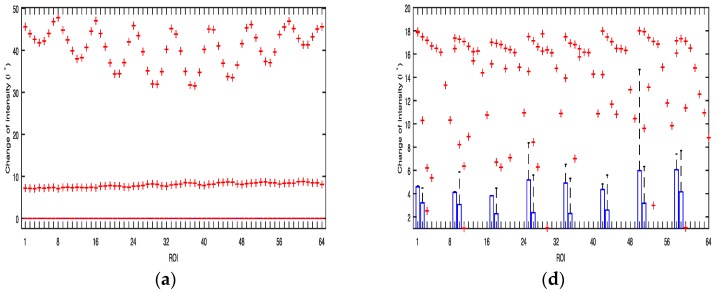

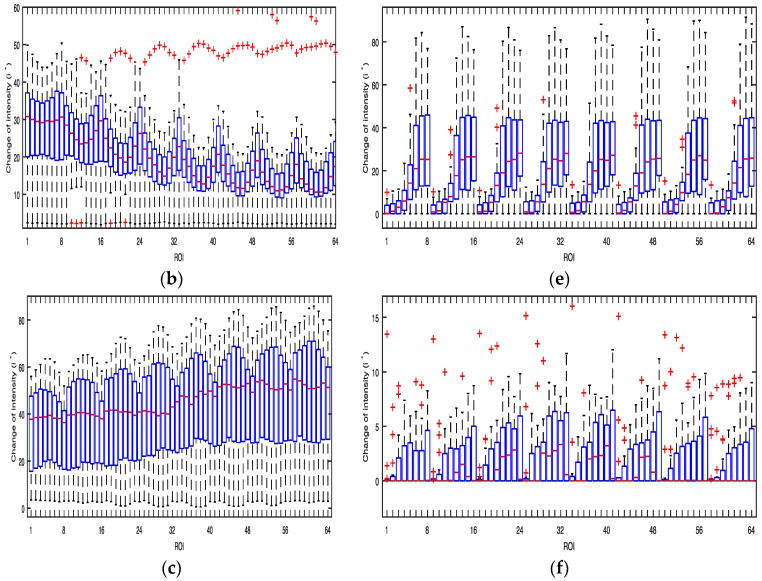
Box plot of the average values of $i^{*}$ of the three color bands obtained from the 64 ROIs of the Galaxy S6 and the Galaxy Note 3 from 30 subjects when the flashlight was on: (**a**) Red (Galaxy S6); (**b**) Green (Galaxy Note 3); (**c**) Blue (Galaxy Note 3); (**d**) Red (Galaxy Note 3); (**e**) Green (Galaxy Note 3); (**f**) Blue (Galaxy Note 3).

Figure 5 shows boxplots with the median calculated by the average values of $i^{*}$ of the three color bands obtained from the 64 ROIs of the Galaxy S6 when the flashlight was on. The lower boundary of the box indicates the 25th percentile, a line within the box marks the median, and the upper boundary of the box indicates the 75th percentile. Whiskers (error bars) above and below the box indicate the 90th and 10th percentiles. Therefore, the area of the blue box is an indication of the spread. Red crosses represent the outliers. In the case of the Galaxy S6, harmonics of green and blue color bands provide a stronger PPG signal than the others while most ROIs of a red color band have low $i^{*}$ values. In the case of the Galaxy Note 3, 1–3 or 6–8 ROIs and their harmonics of all color bands provide strong PPG signals. The average values of $i^{*}$ were found to be highest in blue and green color bands as shown in Table 2.

### 3.2. PPG Signal Quality Analysis with Motion Artifacts

Motion artifacts are known to distort PPG recordings, causing erroneous estimation of HR and respiratory rate. There are three distinct sources of artifacts that can impair PPG recordings: environmental, physiological, and experimental artifacts, which are due to power interference surrounding the body, other physiological signals, and experimental conditions. We investigated the signal quality among the red, green, and blue color bands for each of the pixel regions for all three devices with hand shaking. The participants sat in a chair with their left hand on a desk, with the sensors and instruments required for conventional laboratory measurements attached to the body and left index finger. Then, the subjects were asked to hold the smartphones in their right hand, keeping their right index finger on the camera with the front of the screen vertical on a firm cushion on their knee. The subjects were asked to grip the smartphone sufficiently firmly to ensure that the finger-smartphone contact was constant despite the existence of motion [11].

After baseline recording for 1 minute without any movement, motion artifacts were induced by movement of the subject’s hand in both the horizontal and the vertical directions. Subjects were instructed to introduce motion artifacts horizontally and vertically for specific time intervals. For example, if a subject was instructed to perform left-right movements at 2 Hz or 3 Hz to distinguish between hear rate and motion frequency. We observed that the spectrum by motion artifacts was between 2 Hz and 3 Hz. The shaking intensity was such that visible noise was seen on the recorded PPG waveform. In this period, there were two motion artifact conditions: horizontal motion artifact (HMA) and vertical motion artifact (VMA), and one baseline (BL) condition. In the HMA and the VMA, the experimenter shook the cushion horizontally and vertically, respectively. In the BL, the experimenter simply held the cushion motionless. The experiments of the two motion artifact conditions were conducted on the three smartphones.

**Figure 6:**
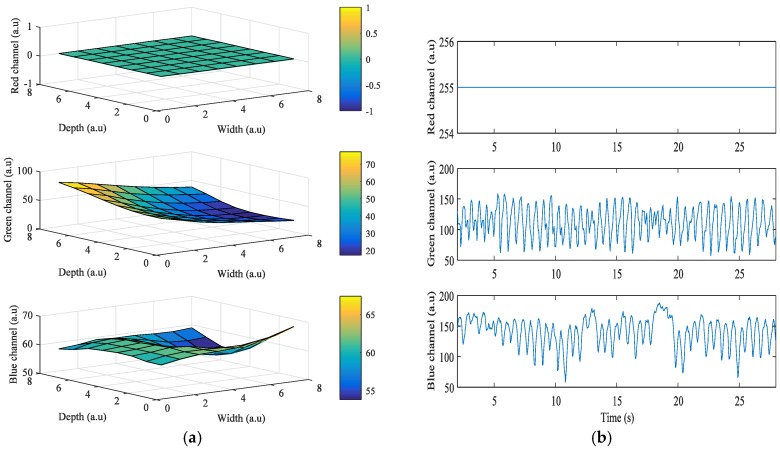
Example of quality analysis of PPG signals obtained from a subject using the Galaxy S6 with horizontal movements: (**a**) intensity change distribution; (**b**) intensity of PPG signal.

Figure 6 shows the results obtained from the Galaxy S6 while shaking the cushion horizontally when the flashlight was on. In the figure, the signal is unstable under the influence of the motion artifact. We measured the red, green, and blue light PPGs using a smartphone while adding motion artifacts. Even though a motion artifact can affect the signal, as ROIs got closer to the flashlight, i^*^ of the green and blue color bands got higher. As shown in Figure 5b, $i^{*}$ of the red, green, and blue color bands were 0, 77.19, and 67.39, respectively. Even though the PPG signals were slight affected by the motion artifact, the heart rate can be measured from the signals of the green and blue color bands. We observed it in other smartphone models.

**Figure 7:**
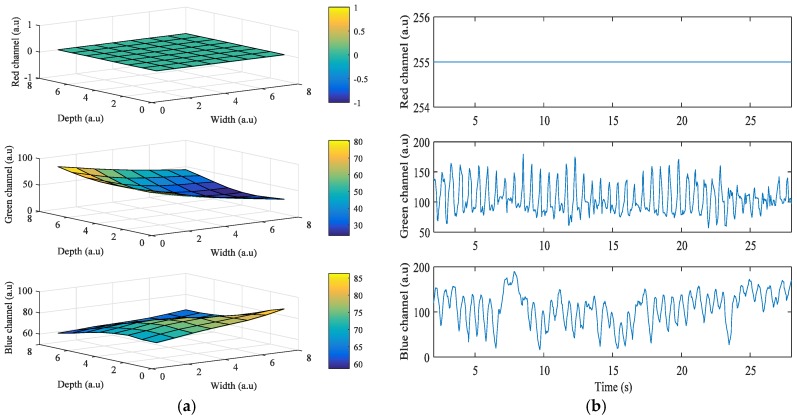
Example of quality analysis of PPG signals obtained from a subject using the Galaxy S6 with vertical movements: (**a**) intensity change distribution; (**b**) intensity of PPG signal.

Figure 7 shows the results obtained from the Galaxy S6 while shaking the cushion vertically when the flashlight was on. In the figure, the signals of the green and blue color bands were stable under the influence of the motion artifact. Because the signal of the blue color band is sometimes unstable, the accuracy of the heart rate estimated from the signal of the green color band is higher than that from the signal of the blue color band. In addition, after reconstructing the MNA contaminated data segments, accurate heart rates and respiratory rates can be measured [6]. We observed it in other smartphone models.

**Figure 8:**
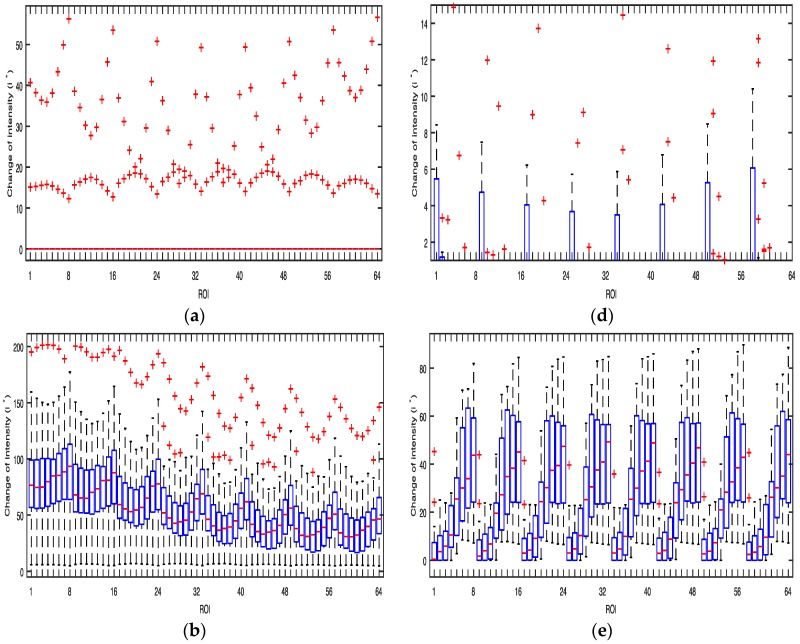

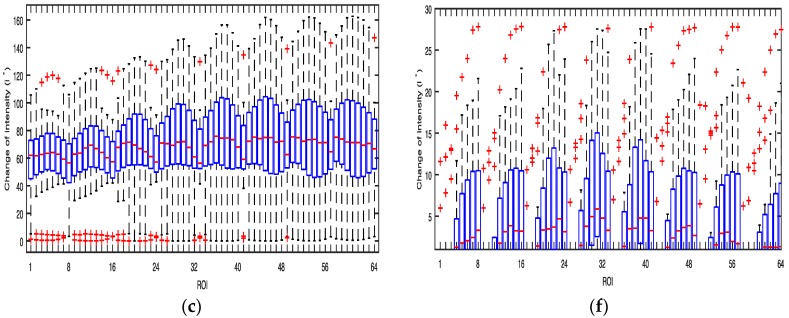
Box plot of the average values of $i^{*}$ of the three color bands obtained from the 64 ROIs of the Galaxy S6 and the Galaxy Note 3 from 30 subjects while shaking the cushion horizontally: (**a**) Red (Galaxy S6); (**b**) Green (Galaxy S6); (**c**) Blue (Galaxy S6); (**d**) Red (Galaxy Note 3); (**e**) Green (Galaxy Note 3); (**f**) Blue (Galaxy Note 3).

**Figure 9:**
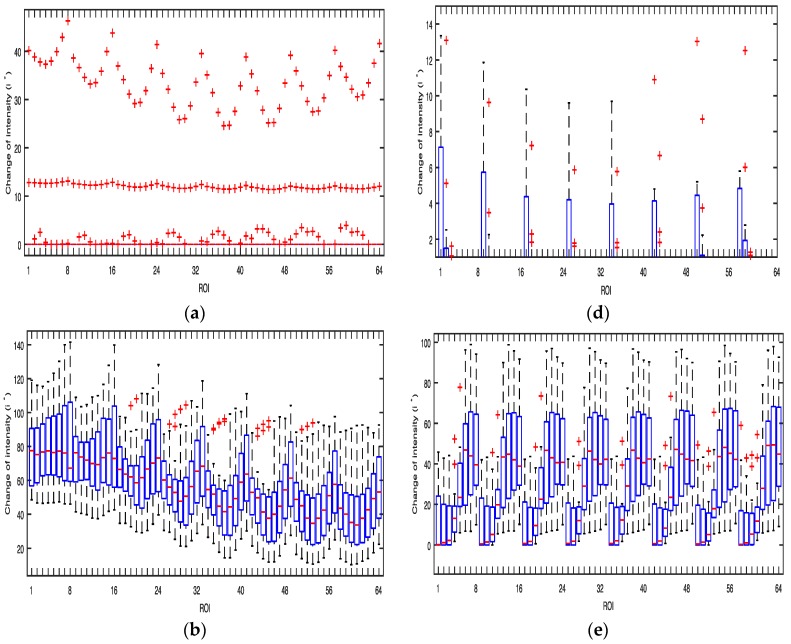

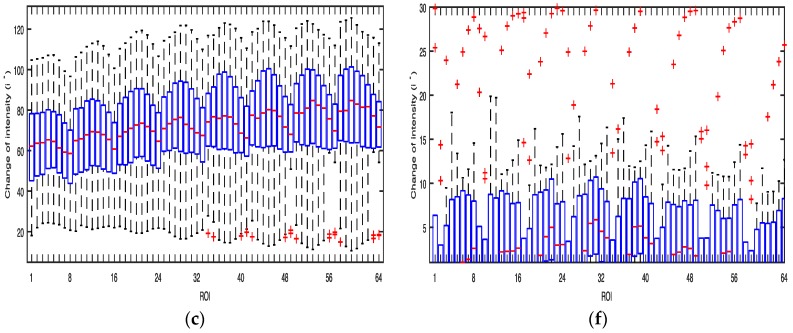
Box plot of the average values of $i^{*}$ of the three color bands obtained from the 64 ROIs of the Galaxy S6 and the Galaxy Note 3 from 30 subjects while shaking the cushion vertically: (**a**) Red (Galaxy S6); (**b**) Green (Galaxy S6); (**c**) Blue (Galaxy S6); (**d**) Red (Galaxy Note 3); (**e**) Green (Galaxy Note 3); (**f**) Blue (Galaxy Note 3).

Figure 8 and Figure 9 show boxplots with the median calculated by the average values of $i^{*}$ of the three color bands obtained from the 64 ROIs of the Galaxy S6 and the Galaxy Note 3 while shaking the cushion horizontally and vertically, respectively, when the flashlight was on. The boxplots of Figure 8 and Figure 9 are similar to those of Figure 5. The average values of $i^{*}$ was found to be highest in blue and green color bands as shown in Table 3.

### 3.3. PPG Signal Quality Analysis with Fingertip Pressure Force

**Figure 10:**
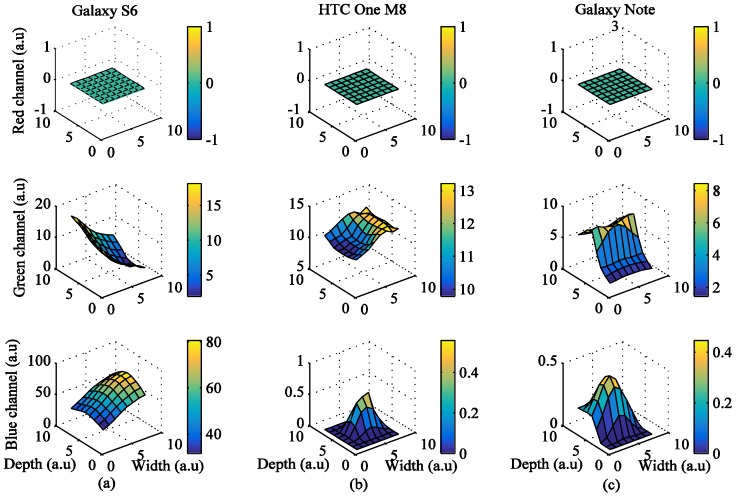
Intensity change distribution of PPG signals with a fingertip pressure force: (**a**) Galaxy S6; (**b**) HTC One M8; (**c**) Galaxy Note 3.

**Figure 11:**
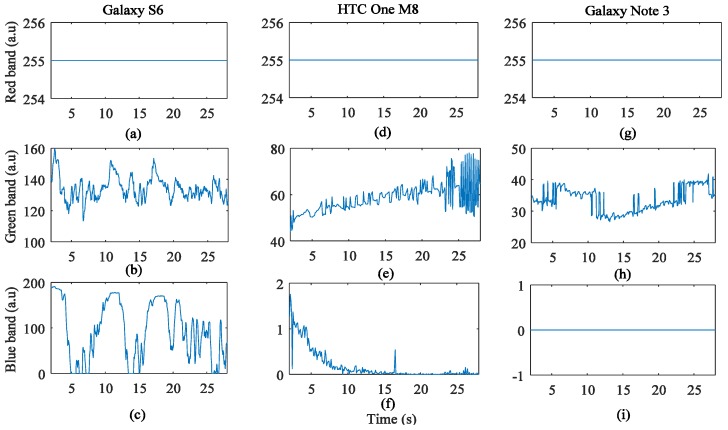
PPG signals with a fingertip pressure force: (**a**) Signal of the red color band of Galaxy S6; (**b**) Signal of the green color band of Galaxy S6; (**c**) Signal of the blue color band of Galaxy S6; (**d**) Signal of the red color band of HTC One M8; (**e**) Signal of the green color band of HTC One M8; (**f**) Signal of the blue color band of HTC One M8; (**g**) Signal of the red color band of Galaxy Note 3; (**h**) Signal of the green color band of Galaxy Note 3; (**i**) Signal of the blue color band of Galaxy Note 3.

All subjects were instructed to put the index fingertip on the smartphone camera lens with different contacting forces over a range between 0.2 N and 1.8 N. This modification was made in consideration of the nature of the present experiment, that is, adding motion artifact [12]. However, it is possible that the finger-phone contact pressure was increased, which could in turn cause a local decrease in arteriolar pressure. In this case, it was observed that the pulse wave disappears when the finger is pressed very firmly against the smartphone image sensor. Figure 10 and Figure 11 show the results of all color bands with a fingertip pressure force on the camera lens. From these figures, HR and respiratory rate cannot estimated from these signals.

### 3.4. PPG Signal Quality Analysis When the Flashlight is Off

**Figure 12:**
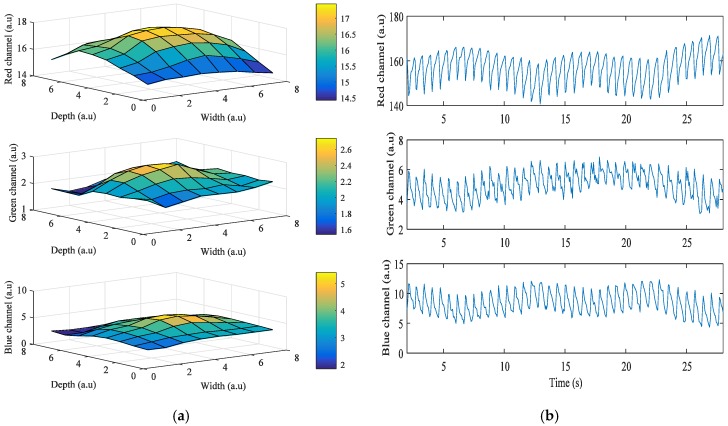
Example of quality analysis of PPG signals obtained from a subject using the Galaxy S6 with flash off: (**a**) intensity change distribution; (**b**) intensity of PPG signal.

**Figure 13:**
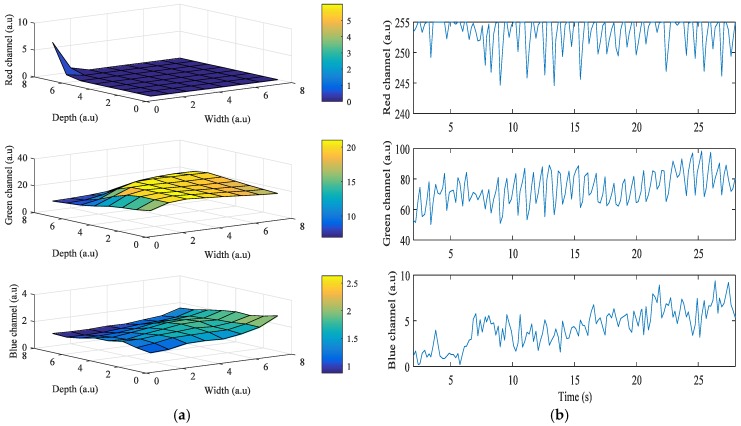
Example of quality analysis of PPG signals obtained from a subject using HTC One M8 with the flash off: (**a**) intensity change distribution; (**b**) intensity of PPG signal.

**Figure 14:**
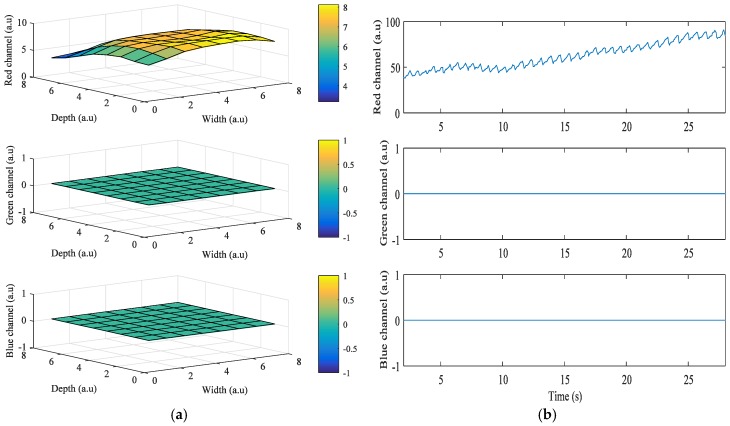
Example of quality analysis of PPG signals obtained from a subject using the Galaxy Note 3 with flash off: (**a**) intensity change distribution; (**b**) intensity of PPG signal.

When the LED flash is off, a change of intensity of the signals of the three color bands was observed in most smartphone models except the Galaxy Note 3. This change of intensity depended on the type of smartphone. Figure 12, Figure 13 and Figure 14 show the results obtained from all smartphones when the flashlight was off. From the figures, the change of intensity was observed mainly in the center of images. In the case of the Galaxy S6, the intensities in the signals of all of the color bands were strongly detected without the flashlight of the smartphone. In the case of the HTC One M8, the heart rate can be measured only from the signals of the green or red color band while the heart rate can be measured from only the signals of the red color band in the case of Galaxy Note 3.

**Figure 15:**
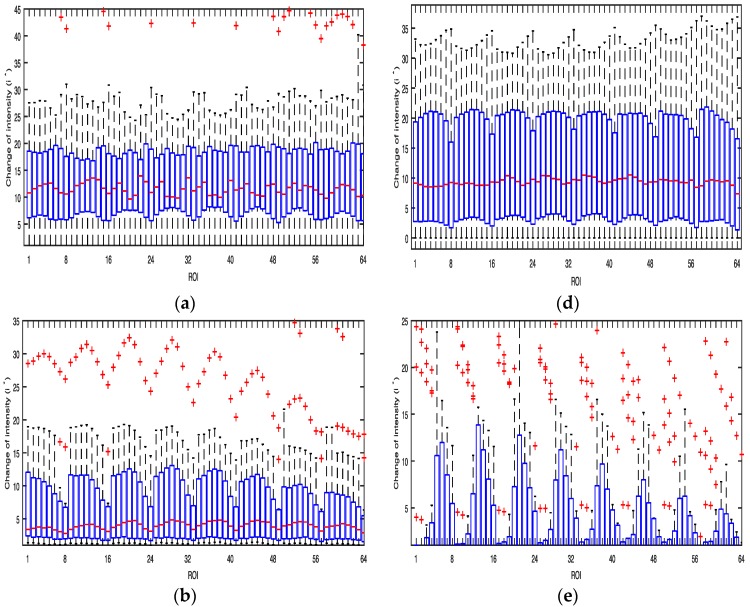

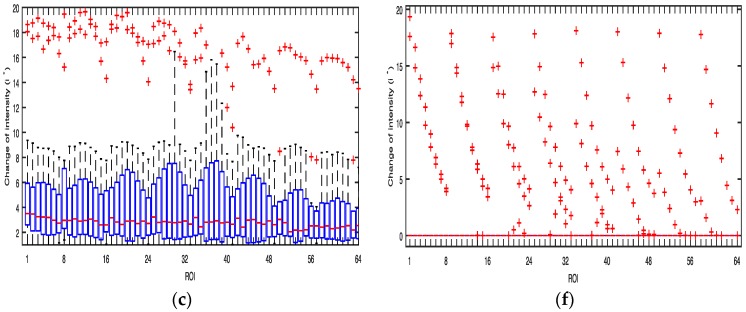
Box plot of the average values of $i^{*}$ of the three color bands obtained from the 64 ROIs of the Galaxy S6 and the Galaxy Note 3 from 30 subjects with flash off: (**a**) Red (Galaxy S6); (**b**) Green (Galaxy S6); (**c**) Blue (Galaxy S6); (**d**) Red (Galaxy Note 3); (**e**) Green (Galaxy Note 3); (**f**) Blue (Galaxy Note 3).

Figure 15 shows boxplots with the median calculated by the average values of $i^{*}$ of the three color bands obtained from the 64 ROIs of the Galaxy S6 and the Galaxy Note 3 when the flashlight was off. The average values of $i^{*}$ was found to be highest in red and green color bands as shown in Table 4.

### 3.5. PPG Signal Quality Analysis of the ROI

**Figure 16:**
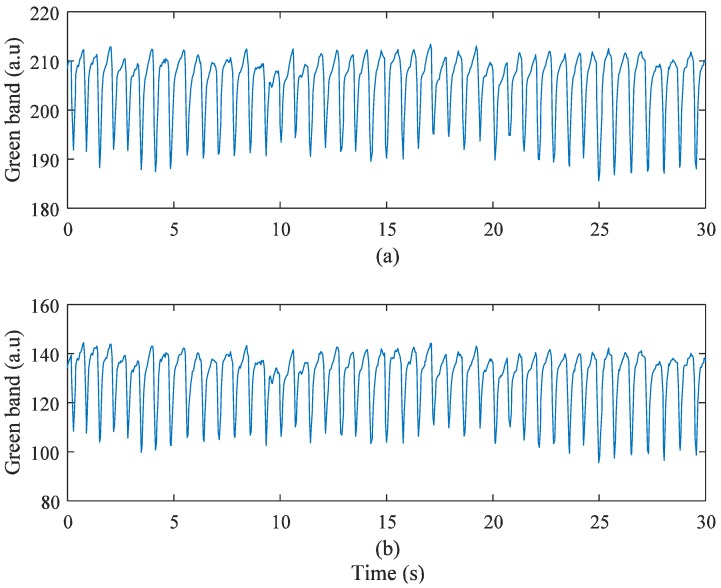
Galaxy S6 with LED On: (**a**) all regions; (**b**) selected dominant region.

**Figure 17:**
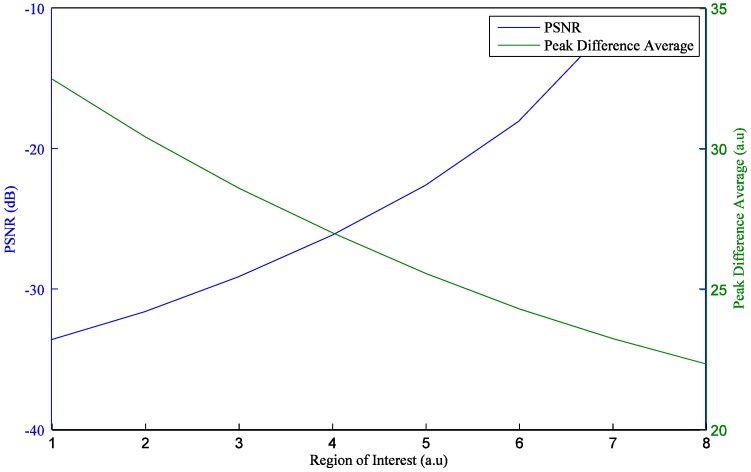
PSNR and i^*^ with ROIs.

When acquiring PPG signals from a smartphone camera, instead of a series of full-size images, this downsized image, namely, the ROI, requires less computational resources and produces more reliable PPG signals [13,14]. Proper selection of the ROI is one of important steps to collect reliable signals. There are two significant advantages to employing an optimized ROI. One is that the computing load is decreased by reducing the image size used to extract the PPG signal. The other is that stronger and more robust PPG signals are obtained by removing the noisy regions. In the experiments, the PPG signal of full-size images was compared with that of ROIs. The ROIs were selected by the optimal ROI determination method [15], which recursively splits regions to locate the region that produces the strongest PPG signal. Figure 16 shows the intensity changes of the full-size images and selected ROIs obtained from the Galaxy S6. When the PPG signals of the full-size images and the selected ROIs were obtained, average values of $i^{*}$ were 22.36 and 32.49, respectively. In other words, $i^{*}$ of the selected ROIs is 45.3% higher than that of full-size images. Figure 17 shows $i^{*}$ and the peak signal-to-noise ratio (PSNR). In the figure, the wider the ROI, the lower $i^{*}$ gets. The wider the ROI, the higher the PSNR gets. In other words, while intensity of the selected ROIs is stronger than that of full-size images, PPG signals obtained from the selected ROIs can be more affected by motion artifacts.

## 4. Conclusions and Discussion

We have investigated signal quality among the red, green, and blue color bands to determine the best signal quality by the largest amplitude values for all three devices under different conditions. We have evaluated the quality of PPG signals obtained from smartphones when the flashlight was on and off. We found that the region closest to the flashlight in most cases resulted in a higher signal quality, which is logical and expected. When the LED flash was off, only the signal of the red color band in all three devices was observed as having a change of intensity, while signals of the green and blue color bands in the Galaxy Note 3 were not observed as having a change of intensity.

The intensity change of a green color band is strongest in the HTC One M8 and the Galaxy Note 3. However, intensity change of a blue color band is strongest in the Galaxy S6. Each smartphone has different image sensors. The Samsung Galaxy S6 has the 16-megapixel Sony Exmor RS IMX240 sensor, which is the same sensor used in the Galaxy Note 4. The HTC One M8 has 4-megapixel OmniVision OV4688 which is a 1/3-inch CMOS sensor. The camera of the Galaxy Note 3 is a Sony IMX135 Exmor RS of 12.8 megapixels. In darker environments, images obtained from the IMX240 sensor of the Galaxy S6 can contain a significant blue tint, which results in strong intensity changes of the blue color band. Even though strong intensity changes of the blue color band were observed in the Galaxy S6, the signals of the green color band obtained from all three devices were stable under conditions except fingertip pressure force conditions.

We also evaluated the quality of the PPG signals obtained from the smartphones with motion artifacts and fingertip pressure force. Even though the signals of all color bands were unstable under the influence of a motion artifact, heart rate can still be estimated using several bandpass filters to cut out a specified frequency band. In addition, after reconstructing the MNA contaminated data segments, accurate heart rates and respiratory rates can be estimated.

Furthermore, we have compared not only the intensity changes of the PPG signals of the full-size images with those of the ROIs, but also the PSNR of PPG signals of the full-size images with that of the ROIs. While the intensity changes of the full-size images were lower than those of selected ROIs, the PSNR of the full-size images was higher than that of the selected ROIs. We hope that our results will help to achieve robust measurements especially in ambulatory settings, where motion-induced artifacts are likely to be a significant issue.

## Figures and Tables

**Table 1:** Experimental conditions.

No.	Conditions
1	Red channel, LED on
2	Green channel, LED on
3	Blue channel, LED on
4	Red channel with horizontal waving, LED on
5	Green channel with horizontal waving, LED on
6	Blue channel with horizontal waving, LED on
7	Red channel with vertical waving, LED on
8	Green channel with vertical waving, LED on
9	Blue channel with vertical waving, LED on
10	Red channel with pressure, LED on
11	Green channel with pressure, LED on
12	Blue channel with pressure, LED on
13	Red channel, LED off
14	Green channel, LED off
15	Blue channel, LED off

**Table 2:** The average values of $i^{*}$ of the three color bands obtained from 64 ROIs when the flashlight was on.

Color Band	Galaxy S6	HTC One M8	Galaxy Note 3
Red	2.77 ± 9.96	18.7 ± 27.12	4.11 ± 11.46
Green	26.39 ± 20.62	35.05 ± 19.47	17.74 ± 14.83
Blue	41.07 ± 21.34	7.51 ± 7.4	1.97 ± 2.87

**Table 3:** The average values of $i^{*}$ of the three color bands obtained from 64 ROIs while shaking the cushion horizontally and vertically when the flashlight was on.

Motion Artifacts	Color band	Galaxy S6	HTC One M8	Galaxy Note 3
	Red	2.97 ± 9.4	13.42 ± 21.14	0.86 ± 2.03
HMA	Green	36.57 ± 69.08	61.07 ± 32.38	24.89 ± 15.59
	Blue	67.21 ± 35.21	7.92 ± 9.07	5.2 ± 35.32
	Red	2.66 ± 8.52	8.06 ± 15.08	2.1 ± 6.75
VMA	Green	65.91 ± 21.89	49.78 ± 28.62	29.91 ± 19.7
	Blue	71.74 ± 27.83	8.58 ± 8.67	7.61 ± 27.95

**Table 4:** The average values of $i^{*}$ of the three color bands obtained from 64 ROIs when the flashlight was on.

Color Band	Galaxy S6	HTC One M8	Galaxy Note 3
Red	34 ± 0.82	5.19 ± 0.25	22.85 ± 0.11
Green	20.3 ± 32.37	16.1 ± 0.18	6.79 ± 0.15
Blue	2.87 ± 9.93	11.2 ± 0	9.3 ± 0
